# The Anti-Inflammatory and Antioxidant Potential of Pistachios (*Pistacia vera* L.) In Vitro and In Vivo

**DOI:** 10.3390/nu9080915

**Published:** 2017-08-22

**Authors:** Irene Paterniti, Daniela Impellizzeri, Marika Cordaro, Rosalba Siracusa, Carlo Bisignano, Enrico Gugliandolo, Arianna Carughi, Emanuela Esposito, Giuseppina Mandalari, Salvatore Cuzzocrea

**Affiliations:** 1 Department of Chemical, Biological, Pharmaceutical and Environmental Science, University of Messina, Viale Ferdinando Stagno D’Alcontres 31, 98166 Messina, Italy; ipaterniti@unime.it (I.P.); dimpellizzeri@unime.it (D.I.); cordarom@unime.it (M.C.); rosiracusa@gmail.com (R.S.); cbisignano@unime.it (C.B.); egugliandolo@unime.it (E.G.); eesposito@unime.it (E.E.); gmandalari@unime.it (G.M.); 2 American Pistachio Growers, 9 River Park Pl E, Fresno, CA 93720, USA; carughia@smccd.edu; 3 Department of Pharmacological and Physiological Science, Saint Louis University School of Medicine, 1402 South Grand Blvd, St. Louis, MO 63104, USA

**Keywords:** pistachio, oxidative stress, inflammation, paw edema

## Abstract

Several reports have demonstrated the effectiveness of pistachio against oxidative stress and inflammation. In this study, we investigate if polyphenols extracts from natural raw shelled pistachios (NP) or roasted salted pistachio (RP) kernels have anti-inflammatory and antioxidant properties at lower doses than reported previously, in both in vitro and in vivo models. The monocyte/macrophage cell line J774 was used to assess the extent of protection by NP and RP pistachios against lipopolysaccharide (LPS)-induced inflammation. Moreover, antioxidant activity of NP and RP was assessed in an in vivo model of paw edema in rats induced by carrageenan (CAR) injection in the paw. Results from the in vitro study demonstrated that pre-treatment with NP (0.01, 0.1 and 0.5 mg/mL) and RP (0.01 and 0.1 mg/mL) exerted a significant protection against LPS induced inflammation. Western blot analysis showed NP reduced the degradation of IκB-α, although not significantly, whereas both NP and RP decreased the TNF-α and IL-1β production in a dose-dependent way. A significant reduction of CAR-induced histological paw damage, neutrophil infiltration and nitrotyrosine formation was observed in the rats treated with NP. These data demonstrated that, at lower doses, polyphenols present in pistachios possess antioxidant and anti-inflammatory properties. This may contribute toward a better understanding of the beneficial health effects associated with consumption of pistachios.

## 1. Introduction

Over the past decade, numerous studies have shown that classes of natural substances derived from higher plants are potentially interesting for therapeutic interventions in various inflammatory diseases [1]. Frequently, treating inflammation with analgesics, non-steroidal anti-inflammatory drugs, and corticosteroid leads to side effects such as gastric discomfort, hypersensitivity reactions, gastric erosion, diabetes mellitus and increased susceptibility to infection [2]. Therefore, it is time to consider plants as possible remedies. Most members of the pistachio genus have chemical and therapeutic similarities. The fruits, nuts, resin and leaves of *Pistacia lentiscus* are used for the treatment of eczema, throat infections, asthma, kidney stones, diarrhea and stomach ache, with astringent, antipyretic, anti-inflammatory, antibacterial, antiviral, pectoral and stimulating properties [3,4,5,6].

In addition, pistachio tree nut has been reported to cause IgE-mediated allergic reactions, comparing three different extracts from raw, roasted, and steam-roasted pistachio nut treatments. The most significant finding of this study was the successful reduction of IgE-binding by pistachio extracts using steam-roast processing without any significant changes in sensory quality of product [7]. The extracts from galls of *P. integerrima* are known to have expectorant, bronchodilator, antiemetic, appetizer, diuretic and antirheumatic effects [8]. The galls of *P. terebinthus*, a small tree from Mediterranean countries, has been used for hip pain gout and rheumatisms [9]. *P. vera* L. (Anacardiaceae family) is a high value product, widely consumed globally because of its nutritional characteristics and health benefits [10]. The United States is considered the second largest producer of pistachios after Iran [11]. There are currently several pistachio bioavailability studies available in the literature [12,13,14,15,16]. One of our previous studies has shown that polyphenols from Natural raw shelled pistachios (*Pistacia vera* L.) (NP) and roasted salted pistachio (RP) kernels were bioaccessible in the upper gastrointestinal tract during simulated human digestion: more than 90% of the total polyphenols were released in the gastric compartment, with virtual total release in the duodenal phase [17]. Anti-inflammatory effects of pistachio nut and anti-inflammatory activity of its components have been the subject of numerous studies in recent years. These effects have been demonstrated in various animal models of acute inflammation such as paw edema [4,8,18,19], LPS-induced inflammation [20], and chronic inflammation models such as colitis [21,22,23,24].

Based of this evidence, the purpose of the present study was to investigate if polyphenols extracted from natural raw shelled pistachios (NP) and from roasted salted pistachio (RP) kernels had anti-inflammatory and antioxidant properties, even at lower doses compared to that observed in the literature. We used two different models where inflammation and oxidative stress play a crucial role. In particular, we have induced the inflammation process in both an in vitro model using cultured LPS-stimulated macrophage cells and an in vivo model of carrageenan-induced fist edema in rats, which is a useful model of acute inflammation.

## 2. Materials and Methods

### 2.1. Pistachios

Californian natural raw, shelled pistachios (NP) and roasted salted pistachio (RP) kernels (*Pistacia vera* L.) were kindly provided by the American Pistachio Growers (Fresno, CA, USA). Pistachio polyphenolic extracts were prepared as previously reported [17,25]. Briefly, NPs or RPs (10 g) were extracted five times with *n*-hexane (100 mL) to remove lipids, after which the residues were mixed with 100 mL of methanol/HCl 0.1% (*v/v*), extracted and centrifuged. After four extractions, the residues were dissolved in distilled water (40 mL) and extracted five times with ethyl acetate (40 mL). Polyphenols analysis was performed using an Ascentis Express C18 column (150 × 4.6 mm, 2.7 μm, Ascentis Express, Supelco, Bellefonte, PA, USA). Polyphenols identification in samples of NP and RP is reported in Table 1. As previously reported [20], NP showed higher amounts of total polyphenols (6.7 mg/100 g) compared with RP (6.0 mg/100 g), with significant differences (*p* < 0.05) in the concentration of gallic acid, catechin, epicatechin and isoquercetin. Experimental research on pistachios complied with the Convention on Biological Diversity and the Convention on the Trade in Endangered Species of Wild Fauna and Flora.

### 2.2. In Vitro Study

#### 2.2.1. Cell Culture and Experimental Groups

The monocyte/macrophage cell-line J774-A1 was cultured and a preliminary analysis involved the study of cell viability: 4 × 10^4^ cells were plated (in a volume of 150 µL) in 96-well plates and allowed to adhere for 4 h at 37 °C. Thereafter, the medium was replaced with fresh medium and cells were treated with 4 different concentrations (0.01 mg/mL, 0.1 mg/mL, 0.5 mg/mL, and 1.0 mg/mL) of both NP and RP, to determine the high concentrations with less toxicity on cell viability. Once the high concentrations with less toxicity were determined, we stimulated the cells with LPS (from *Escherichia coli* 1.0 µg/mL) for 24 h [26].

#### 2.2.2. Vital Staining

To assess viability of cell cultures, cells were incubated at 37 °C with 0.2 mg/mL MTT (3-[4,5-dimethylthiazol-2-yl]-2,5 diphenyl tetrazolium bromide) for 1 h. Cell viability was quantified by measurement of optical density at 550 nm (OD550) using a microplate reader [27].

#### 2.2.3. Western Blot Analysis

Extracts of macrophages stimulated for 24 h with LPS were prepared as previous described [28]. Specific primary antibodies anti-iNOS (1:500 Trasduction), anti-COX2 (1:500; Cayman Chemical, Ann Arbor, MI, USA) and anti-IκB-α (1:500; Santa Cruz Biotechnology, Dallas, TX, USA) were used. Membranes were then incubated secondary antibody (1:2000, Jackson ImmunoResearch, West Grove, PA, USA) for 1 h at room temperature. To ascertain that blots were loaded with equal amounts of proteic lysates, they were also incubated in the presence of the antibody β-actin (1:500; Santa Cruz Biotechnology). Signals were detected with an enhanced chemiluminescence detection system reagent. Relative expression of protein bands was quantified by densitometry (optical density [OD] per mm^2^) with ChemiDoc™ XRS+ (Image Lab version 5.2.1 build 11, Bio-Rad Laboratories, Hercules, CA, USA) and standardized to β-actin levels. 

#### 2.2.4. Measurement of Nitrite Levels

Total nitrite levels, as an indicator of nitric oxide (NO) synthesis, were measured in the supernatant as previously described [28].

#### 2.2.5. Measurement of Cytokine Production

The medium samples were mixed prior to their use in TNF-α and IL-1β ELISA assays, according to manufacturer’s details. Absorbance was read at 450 nm and background wavelength correction set at 540 nm or 570 nm.

#### 2.2.6. Determination of Intracellular ROS

Intracellular ROS was detected using the total ROS detection kit as previously shown [29]. After various treatments, the monocyte/macrophage cell-line J774 were trypsinized and then washed twice with 1× washing buffer. Subsequently, the cells were incubated with 5-(and-6)-carboxy-2′,7′-dichlorodihydrofluorescein diacetate (carboxy-H2DCFDA; 10 μM final concentration) at 37 °C in the dark for 30 min. The fluorescence microplate reader detected the light emission. The level of intracellular ROS was expressed as the percentage of the control (nmol/mL).

#### 2.2.7. Determination of Malondialdehyde (MDA) Levels

The monocyte/macrophage cell-line J774 (1 × 105 cells/ well) was seeded in poly-l-lysine-coated six-well plates. The cells were harvested to detect the levels of malondialdehyde (MDA) using the MDA assay kit as previously described.

### 2.3. In Vivo Study

#### 2.3.1. Animals

The study was carried out on Sprague–Dawley male rats (200–230 g, Harlan, Nossan, Italy). Food and water were available ad libitum; the animals were fed with a standard diet. The study was approved by the University of Messina Review Board for the care of animals. Animal care was in compliance with Italian regulations on protection of animals used for experimental and other scientific purposes (D.M.116192) as well as with the EEC regulations (O.J. of E.C. L 358/1 12/18/1986). This study conforms to the “ARRIVE Guidelines for Reporting Animal Research”. Authors declare that the research complies with the commonly-accepted “3Rs”: Replacement, Reduction and Refinement.

#### 2.3.2. Carrageenan-Induced Paw Edema

Paw edema was induced as previously described by subplantar injection of CAR (0.1 mL of a 1% suspension in 0.85% saline) into the right hind paw on rats [30,31]. At the end of the experiment, animals were killed under anesthesia and hind paws were fixed in 10% neutral buffered formalin and embedded in paraffin for both histological and immunohistochemical examinations or stored at −70 °C and used for further analyses.

The volume of paw edema was measured by a plethysmometer (Ugo Basile, Comerio, Varese, Italy) prior to car injection and every hour for 6 h. Edema was expressed as the increase in paw volume (mL) after carrageenan injection relative to the pre-injection value for all animal. Scores are expressed as paw volume difference (mL).

#### 2.3.3. Experimental Groups

First, rats were randomly allocated into the following groups:

(i) CAR group, rats were injected with CAR to induced paw edema (*n* = 10);
(ii) CAR + NP, same as the CAR group and NP (30 mg/kg) was orally administered 30 min before CAR (*n* = 10); and
(iii) CAR + RP, same as the CAR group and RP (30 mg/kg) was orally administered 30 min before CAR (*n* = 10).

The sham-operated group received saline, a vehicle of pistachios, instead of carrageenan (*n* = 10 for all experimental groups).

The dose of 30 mg/kg was chosen based on a previous dose–response experiment that we did in our laboratories, in which rats were treated with 10, 30 and 100 mg/kg of NP or RP and we observed that the dose of 30 mg/kg was the highest dose without toxicity.

#### 2.3.4. Histological Examination of the CAR-Inflamed Hind Paw

Seven-micrometer-thick sections stained with haematoxylin and eosin (H&E) were examinated using light microscopy associated to an Imaging system (AxioVision, Zeiss, Milan, Italy) and scored by two investigators in a blind fashion. The sections were stained with H&E to allow a complete histological analysis that identified the morphological characteristics of the muscle fibers, from 0 to 5, defined as follows: 0 = no inflammation; 1 = mild inflammation; 2 = mild/moderate inflammation; 3 = moderate inflammation; 4 = moderate/severe inflammation; and 5 = severe inflammation [32].

#### 2.3.5. Myeloperoxidase Activity

MPO activity, an index of polymorphonuclear cell accumulation, was determined as previously described [33] in the palm of hind paw tissues. The rate of change in absorbance was measured spectrophotometrically at 650 nm. MPO activity was measured as the quantity of enzyme degrading 1 mM of peroxide 1 minute at 37 °C, and was expressed in units per gram weight of wet tissue.

#### 2.3.6. Immunohistochemistry for Nitroyrosine

Immunohistochemical analysis for nitroyrosine was performed in the palm of hind paw sections as described in previous studies [34]. At the end of the experiment, the tissues were fixed in 10% (*w/v*) PBS-buffered formaldehyde, and 7-μm sections were prepared from paraffin- embedded tissues. After de-paraffinization, endogenous peroxidase was quenched with 0.3% (*v/v*) hydrogen peroxide in 60% (*v/v*) methanol for 30 min. The sections were permeabilized with 0.1% (*w/v*) Triton X-100 in PBS for 20 min. Non-specific adsorption was minimized by incubating the sections in 2% (*v/v*) normal goat serum in PBS for 20 min. Endogenous biotin- or avidin-binding sites were blocked by sequential incubation for 15 min with biotin and avidin (DBA), respectively. Sections were incubated overnight with anti-nitrotyrosine polyclonal antibody (1:500 in PBS (*v/v*)). Sections were washed in PBS and incubated with secondary antibody. Specific labeling was detected with a biotin conjugated goat anti-rabbit IgG and avidin–biotin peroxidase complex (Vector) (D.B.A s.r.l, Milan, Italy). The counter stain was developed with diaminobenzidine (brown color) and nuclear fast red (red background). The photographs obtained (*n* = 5 photos from each sample collected from all animals in each experimental group) were assessed by densitometry using Leica QWin (software version V3, Leica Microsystems, Cambridge, UK). The percentage area of immunoreactivity was expressed as percent of total tissue area.

### 2.4. Materials

Unless otherwise stated, all compounds were obtained from Sigma-Aldrich (St. Louis, MO, USA). All other chemicals were of the highest commercial grade available. All stock solutions were prepared in non-pyrogenic saline (0.9% NaCl, Baxter, Milan, Italy) or 10% dimethyl sulfoxide.

### 2.5. Statistical Analysis

All values are expressed as mean ± SEM. The results were analyzed by one-way ANOVA followed by a Bonferroni post-hoc test for multiple comparisons. A value of *p* ≤ 0.05 was pre-determined as the criterion of significance. The number of animals used for in vivo studies was carried out by G * Power 3 software (Die Heinrich-Heine-Universität Düsseldorf, Düsseldorf, Germany).

## 3. Results

### 3.1. Effect of NP and RP on Cell Viability

**Figure 1:**
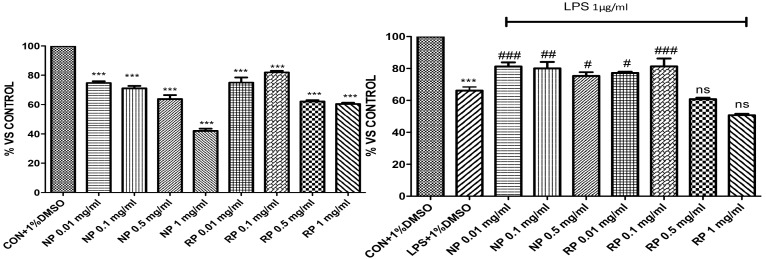
Effect of the pistachios on cell viability. Cell viability was assessed 24 h after treatment with the indicated concentrations (1.0 mg/mL, 0.5 mg/mL, 0.1 mg/mL, and 0.01 mg/mL) of NP and RP, respectively; cell viability was significantly reduced with NP at the highest concentration of 1 mg/mL. NP and RP at 0.5, 0.1, and 0.01 mg/mL lacked cytotoxicity. Moreover, incubation of cells with LPS significantly reduced cell viability compared to the control group, whereas pretreatment with NP at the concentrations of 0.01, 0.1 and 0.5 mg/mL and RP at the concentrations of 0.0 and 0.1 mg/mL significantly limited reduction of cell viability. Data are representative of at least three independent experiments; *** *p* < 0.001 vs. Ctr; ^###^ *p* < 0.001 vs. LPS; ^##^ *p* < 0.01 vs. LPS; ^#^ *p* < 0.05 vs. LPS.

To test the effect on cell viability, J774 cells were incubated with increasing concentrations of NP and RP (from 0.01 mg/mL to 1.0 mg/mL). NP and RP at the high concentration of 1.0 mg/mL reduced cell viability by 55% and 33%, respectively (Figure 1), whereas the concentrations of 0.5 mg/mL, 0.1 mg/mL, and 0.01 mg/mL showed no toxic effects on cell viability, which is around 82% and 77%.

Since the concentration of NP at 1.0 mg/mL induced a high reduction of the cell viability, this concentration was not used in further experiments.

Further, we stimulated cells with LPS to induce inflammatory response, and pre-treated cells with NP and RP. The results obtained showed a significant protective effect against LPS induced inflammatory process in cells pre-treated with NP at the three concentrations used (0.5 mg/mL, 0.1 mg/mL and 0.01 mg/mL), whereas pre-treatment with RP exerted significant protection only at the concentrations of 0.01 mg/mL and 0.1 mg/mL.

### 3.2. Effect of NP and RP on IκB-α Expression

**Figure 2:**
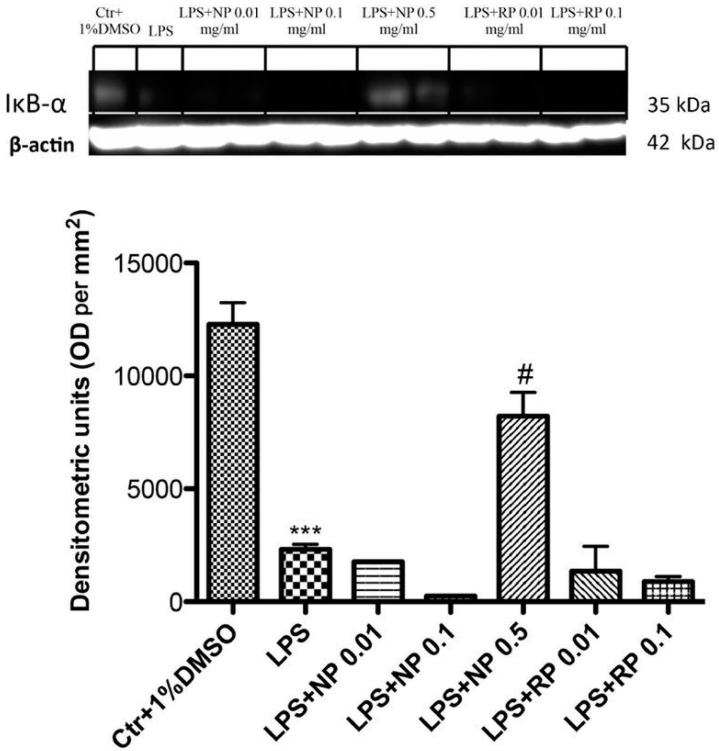
Effect of NP and RP on IκB-α expression. Western blot analysis demonstrated basal levels for IκB-α in the control group, whereas stimulation with LPS significantly induced the degradation of IκB-α levels. Treatments with NP at the concentration of 0.5 mg/mL and RP at 0.1 mg/mL increased the levels of IκB-α, but this protection was not significant. Data are representative of at least three independent experiments; *** *p* < 0.001 vs. Ctr; ^#^ *p* < 0.05 vs. LPS.

To investigate how NP and RP could attenuate the inflammatory process induced by LPS stimulation, we evaluated IκB-α expression. The results obtained showed a basal expression of IκB-α in the cytoplasmic fraction of the control cells, while IκB-α levels significantly decreased after stimulation with LPS (Figure 2). Pre-treatment with NP at the highest tested concentration 0.5 mg/mL and RP at 0.1 reduced IκB-α degradation, although not significantly. No effect was observed with RP and NP at the concentrations of 0.01 mg/mL.

### 3.3. Effect of NP and RP on TNF-α and IL-1β Expression

**Figure 3:**
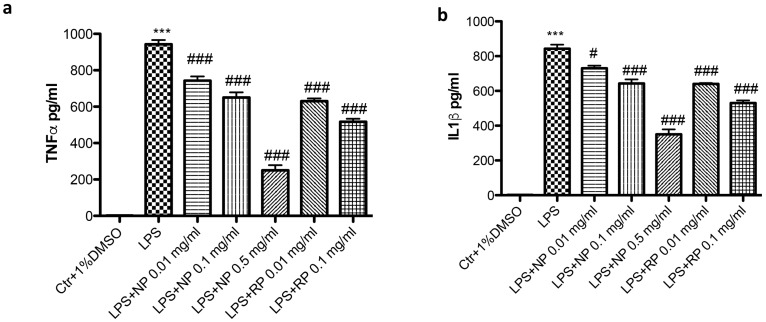
Effect of NP and RP of pro-inflammatory cytokines production. LPS stimulation significantly increased the levels of: TNF-α (**a**); and IL-1β (**b**). Treatments with NP and RP decreased the levels of: TNF-α (**a**); and IL-1β (**b**) in a concentration dependent manner. *** *p* < 0.001 vs. Ctr; ^###^ *p* < 0.001 vs. LPS; ^#^ *p* < 0.05 vs. LPS.

The levels of the pro-inflammatory cytokines TNF-α and IL-1β were evaluated by Elisa kit. An increase in the production of both TNF-α and IL-1β was recorded after LPS stimulation (Figure 3a,b). However, pre-treatment with both NP and RP significantly decreased the levels of TNF-α and IL-1β in a concentration-dependent manner (Figure 3a,b).

### 3.4. Effect of NP and RP on iNOS, COX-2 and Nitrite Expression

**Figure 4:**
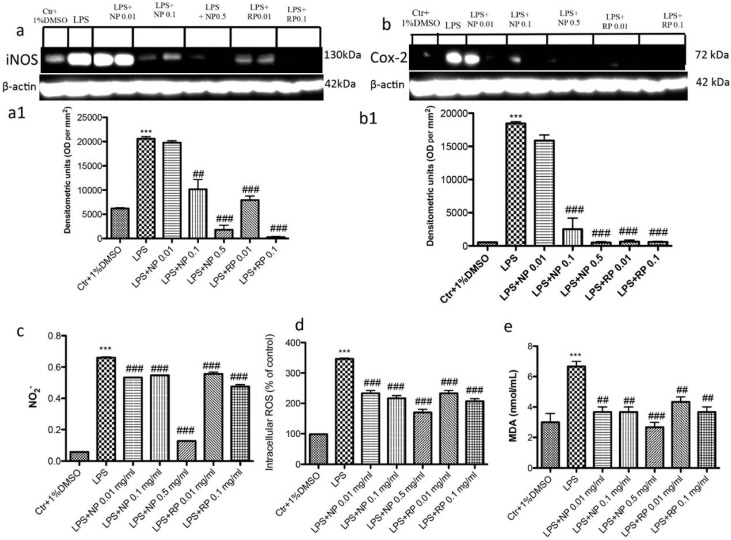
Effect of NP and RP on the expression of iNOS, COX-2 and nitrite levels. Western blot analysis for iNOS and COX-2 demonstrated a significant increased levels after LPS stimulation (**a**,**b**); treatments with NP only at concentrations of 0.1 and 0.5 mg/mL and RP at 0.01 and 0.1 mg/mL significantly reduced iNOS expressions. No protection was observed with NP 0.01 mg/mL. The levels of COX-2 were also reduced with NP treatment at concentrations of 0.1 and 0.5 mg/mL and RP at 0.01 and 0.1 mg/mL. Less protection, but significant, was observed with NP 0.01 mg/mL. Moreover, we analyzed the levels of nitrite production and we observed an increase of nitrite levels after LPS stimulation (**c**), whereas treatments with NP, only at 0.5 mg/mL, significantly reduced nitrite production. Moreover, we determinate the levels of ROS content and MDA production (**d**,**e**), and observed an increase of ROS content and MDA levels after LPS stimulation, whereas treatments with NP and RP reduced ROS and MDA levels at concentration dependent manner. Data are representative of at least three independent experiments; *** *p* < 0.001 vs. Ctr; ^###^ *p* < 0.001 vs. LPS; ^##^ *p* < 0.01 vs. LPS.

To evaluate the nitrosative stress induced by LPS stimulation and the protective role played by pistachios, we performed Western blots for iNOS and COX-2. Basal levels of iNOS were observed in the control groups, whereas LPS stimulation induced a significant increase in iNOS expression (Figure 4a). Pre-treatment with NP at the concentration of 0.1 and 0.5 mg/mL significantly reduced the expression of iNOS, whereas the concentration of 0.01 mg/mL had no significant effect. Pre-treatment with RP at the concentration of 0.1 significantly reduced the expression of iNOS, whereas the concentration of 0.01 mg/mL had no significant effect (Figure 4a).

The COX-2 levels were significantly increased after LPS stimulation, whereas pre-treatment with both NP and RP at all concentrations tested significantly reduced COX-2 levels (Figure 4b).

Moreover, we investigated the levels of nitrite released into the culture medium by Griess reagent. The untreated control group released low levels of NO^2−^, whereas LPS stimulation significantly increased the levels of NO^2−^ production (Figure 4c). Pre-treatment with both NP and RP extracts decreased NO production in a concentration-dependent manner (Figure 4c).

Moreover, to better investigate the antioxidant capacity of NP and RP, we measure the ROS content and the MDA levels (Figure 4d,e, respectively).

The untreated control group released low levels of ROS and MDA, whereas LPS stimulation significantly increased ROS content and the MDA levels (Figure 4d,e, respectively). Pre-treatment with both NP and RP extracts significantly decreased ROS and MDA production in a concentration-dependent manner (Figure 4d,e, respectively).

### 3.5. Effect of NP and RP on the Time-Course of Carrageenan-Induced Paw Edema in Rats

**Figure 5:**
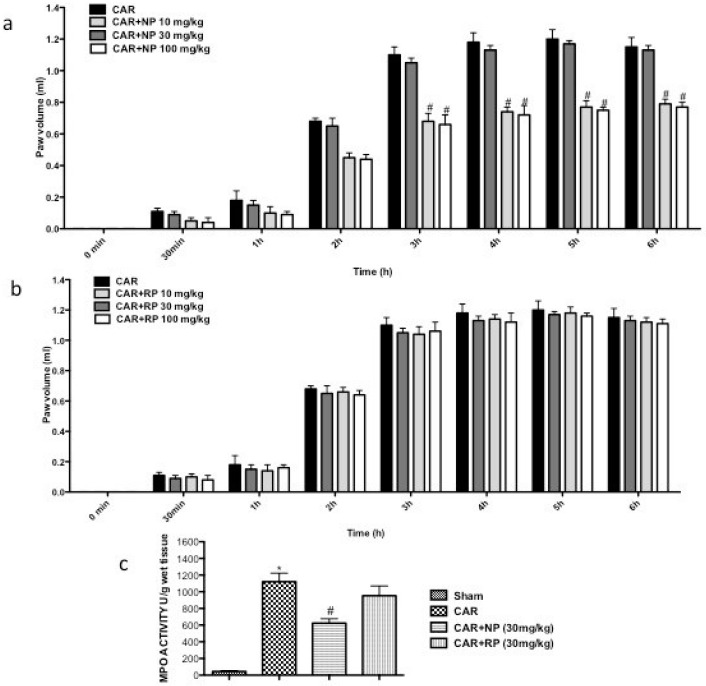
Effect of NP and RP on the time course of carrageenan-induced paw edema. NP and RP were administered orally 30 min before CAR injection. Paw edema was assessed at the time points indicated (**a**,**b**). NP produced significant improvements in the paw edema (measured as paw volume) in comparison to RP administered at the same time point and at the same doses. Moreover, we observed increased levels of MPO after CAR injections and treatments with NP, but no RP significantly reduced levels of MPO (**c**). Values are means ± SEM. * *p* < 0.05 vs. Sham; ^#^ *p* < 0.05 vs. CAR.

Injection of CAR into the sub-plantar region of the right hind paw rapidly induced paw edema in rats, which was maximal after 5 h in CAR injected rats (Figure 5a,b). A significant reduction of the paw edema volume was observed in rats treated with NP at 30 and 100 mg/kg (Figure 5a) compared to the sham group, whereas treatment RP did not significantly affect the paw edema (Figure 5b). Moreover, MPO activity was measured in the palm of hind paw tissues as a marker of neutrophilic infiltration: an increase of MPO activity was found in CAR injected rats (Figure 5c). Administration of NP 30 mg/kg significantly reduced MPO activity, whereas RP at 30 mg/kg did not produce a reduction in neutrophil infiltration in the paw tissues (Figure 5c).

### 3.6. NP Inhibited CAR-Induced Histological Paw Damage and Neutrophil Infiltration

**Figure 6:**
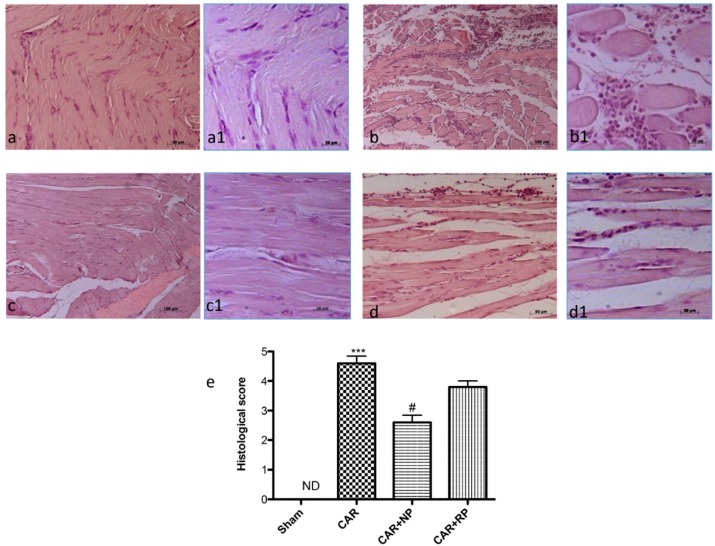
Anti-inflammatory effects of NP histological analysis. Histological evaluation was performed by hematoxylin and eosin staining: (**a**) the control group; (**b**) the intraplantar CAR injection; (**c**,**d**) CAR with NP treatment and CAR with RP treatment, respectively; (**a1**–**d1**) low magnification of the respective panels; and (**e**) histological score for the various treatment groups. The figures are representative of at least three independent experiments. *** *p* < 0.001 vs. Sham; ^#^ *p* < 0.05 vs. CAR.

To assess the anti-inflammatory and antioxidant effects of NP and RP, the palm of hind paw tissues were examined by hematoxylin and eosin staining. While tissue from sham-treated rats showed no histologic alteration and normal fibers (Figure 6a and insert Figure 6a1, see histological score Figure 6e, a disorganized muscle fibers of various shapes and sizes with irregular contours, important amassing of infiltrating inflammatory cells, edema, loss of normal muscle paw architecture, and increased inter-fiber space were evident after CAR injection into the right hind paw (Figure 6b and insert Figure 6b1, see histological score Figure 6e). Muscle fibers of normal appearance, exhibiting some infiltrating inflammatory cells, was observed after NP treatment (Figure 6c and insert Figure 6c1, see histological score Figure 6e). However, treatment with RP failed to ameliorate this damage, in which accumulation of infiltrating inflammatory cells, edema, increased inter-fiber space and disorganization of normal muscle paw morphology were still observed (Figure 6d and insert Figure 6d1, see histological score Figure 6e).

### 3.7. NP Inhibited CAR-Induced Nitrotyrosine Formation

**Figure 7:**
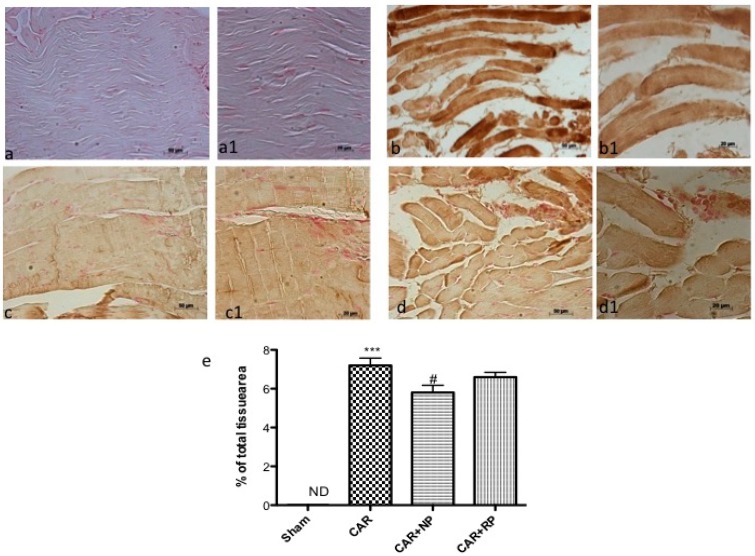
Peroxynitrite production following intraplantar injection of CAR in rat hind paw. Immunohistochemistry for nitrotyrosine showed positive staining in paw tissue sections from CAR-injected rats (**b**). The intensity of nitrotyrosine staining was significantly reduced in the paw from NP-treated rats (**c**); compared to RP-treated rats (**d**). (**a1**–**d1**) low magnification of the respective panels. The figures are representative of at least three independent experiments. *** *p* < 0.001 vs. Sham; ^#^ *p* < 0.05 vs. CAR.

The possible participation of peroxynitrite in reactive oxygen species (ROS)-mediated nociception was evaluated by immunohistochemical detection of nitrated proteins (nitrotyrosine formation). Nitrotyrosine expression was clearly detectable after CAR injection into the hind paw tissue (Figure 7b and insert Figure 7b1, see densitometry analysis Figure 7e). The formation of nitrated proteins was blocked by NP but not by RP (Figure 7c and insert Figure 7c1, see densitometry analysis Figure 7e). This effect better explains the antioxidant effect of NP.

## 4. Discussion

In the literature, there are several studies regarding the beneficial effects of pistachio. In particular, there are studies on carrageenan or LPS-induced acute inflammatory response [4,16], inflammatory bowel disease and colitis [21,35,36,37], cancer [38,39,40], allergic inflammation in asthmatic model [41] and many other experimental models. Furthermore, the antimicrobial properties of polyphenolic fractions obtained from natural raw and roasted salted pistachios have also been evaluated [42,43]. Several studies have reported the potent antioxidant, anti-inflammatory and anti-apoptotic potential of pistachio [44,45,46,47,48].

Therefore, the purpose of our research was to demonstrate for the first time that polyphenols extracted from natural raw shelled pistachios (NP) and from roasted salted pistachio (RP) kernels possessed antioxidant and anti-inflammatory properties at doses lower than those found in the literature.

In the in vivo study, we have demonstrated that the polyphenols-rich extract obtained from NP significantly reduced the paw edema in rats, with a decrease in MPO activity that a marker of neutrophilic infiltration. No significant effect was detected when using the RP polyphenols-rich extract. This could be due to the higher levels of bioactive compounds identified in NP compared with RP [17]. Furthermore, the synergistic interaction amongst the polyphenols identified in NP could enhance its bioactivity. The strong antioxidant effect of catechin, whose concentration is double in NP compared to RP (Table 1), has been widely reported [49]. The concentration of epicatechin and isoquercetin is also significantly higher in NP compared to RP (Table 1): we believe that these compounds contribute to the strong antioxidant and anti-inflammatory properties of the extract.

Various studies on pistachio have clearly indicated a crucial role played by NF-κB in the gene regulation associated to proteins or mediators of inflammation [4,50]. For example, it has been previously shown that NP is able to inhibit the degradation of IKB-alpha and the consequent NFKB translocation in the nucleus [16]. Furthermore, it has been shown that the hydrophilic extract from Sicilian *Pistacia* L. is capable of influencing redox-sensitive signal transduction pathways thus modulating NF-κB activity and finally decreasing the regulation of iNOS expression, COX-2 and TNF-α [16,20,44,45].

Therefore, we sought to evaluate where NP and RP polyphenols-rich extracts had effects on the expression of these proteins, even at lower doses than reported previously. Treatment with NP and RP significantly reduced TNF-α and IL-1β levels, and attenuated the production of iNOS. These observations are in agreement with previous studies evaluating the anti-inflammatory properties of plant materials and molecules of bioactives [51,52,53,54]. Thus, the antioxidant and anti-inflammatory properties of polyphenols from pistachios could be attributed to the reduction of the nitrosative stress and subsequent formation of NO. This was reported earlier, but at doses much higher than those used in the present study.

Taken together, our data demonstrated that polyphenols from pistachios, at lower doses that reported in literature, were able to protect from oxidative stress reducing the expression of markers of nitrosative stress such as iNOS, COX2 and NO formation.

## 5. Conclusions

In conclusion, we have demonstrated that the bioactives present in pistachios exhibit some antioxidants and anti-inflammatory properties at lower doses in vitro and in vivo, suggesting a potential therapeutic use of these natural products. However, in-depth studies and more appropriate models are warranted on the mechanisms involved.

## Figures and Tables

**Table 1:** Flavonoids and phenolic acids in NP and RP.

Compound	NP	RP
Gallic acid	1.18 ± 0.12 *	2.05 ± 0.24 *
Protocatechuic acid	0.88 ± 0.04	0.96 ± 0.16
Chlorogenic acid	-	0.18 ± 0.02
Catechin	2.19 ± 0.20 *	0.95 ± 0.06 *
Epicatechin	0.15 ± 0.01 *	0.08 ± 0.02 *
Eriodictyol-7-*O*-glucoside	0.01 ± 0.00	0.03 ± 0.00
Quercetin-3-*O*-rutinoside	0.58 ± 0.04	0.55 ± 0.04
Isoquercetin	1.52 ± 0.22 *	0.81 ± 0.10 *
Daidzein	-	0.15 ± 0.01
Eriodictyol	0.06 ± 0.02	0.05 ± 0.01
Luteolin	0.18 ± 0.03	0.22 ± 0.11

-, trace of detected compound; NP, natural raw pistachio extract; RP, roasted salted pistachio extract. Values are expressed as mg per 100 g and represent the average of triplicate measurements ± SD. Differences among concentration of polyphenols in NP and RP were assessed by analysis of variance followed by the Tukey pairwise comparison. Two-sample *t* tests (two-tailed) were used. The regression values were considered statistically significant at *p* < 0.05. * indicates significant differences.
